# Changes to Drug-Coated Balloon Reimbursement Affect Peripheral Arterial Disease Patient Care: A Single-Center Experience

**DOI:** 10.7759/cureus.23514

**Published:** 2022-03-26

**Authors:** Arthur M Samia, George L Adams

**Affiliations:** 1 Dermatology, University of Florida, Gainesville, USA; 2 Interventional Cardiology, University of North Carolina (UNC) REX Hospital, Raleigh, USA

**Keywords:** drug-coated balloon, peripheral arterial diseases, interventional cardiology, hospital reimbursement rate, pass-through code, bare-metal stents, drug-eluting stents

## Abstract

Introduction: Balloon angioplasty (BA) and stenting have long been the mainstays of endovascular therapy in peripheral arterial disease (PAD). However, the rise of drug-coated balloons (DCBs) has revolutionized care in recent years, with multiple clinical trials showing superiority over BA in maintaining primary patency and freedom from target lesion revascularization (TLR). With the recent drop of the add-on payment for DCBs, a barrier for their use and consequently reduced therapy adoption in PAD might arise. We assessed if this affected physicians' behavior and hospital administration towards stocking and using DCBs.

Methods: This single-center, retrospective study evaluated DCB utilization in 2017 versus 2018. Data were collected in two groups: 1) July 1, 2017, to December 31, 2017 - with pass-through code (PTC) - prior medical billing reimbursement - and 2) January 1, 2018, to June 30, 2018 - without PTC - markedly reduced reimbursement. Patients treated for superficial femoral artery (SFA) or popliteal artery (POP) disease were included. The study aimed to determine changes in DCB utilization between the years with and without PTC, and we investigated the treatments that have replaced DCBs. Additionally, we aimed to collect data on readmissions and procedure costs compared to national data.

Results: From July through December 2017, 350 DCBs were used in 209 patients (1.675 DCBs per patient), while from January through June 2018, 256 DCBs were used in 180 patients (1.422 DCBs per patient) - a 15.07% reduction in DCBs per patient. The detailed numbers of DCB-treated patients were presented as fractions of total interventions in the groups with and without PTC.

Conclusion: The findings of this study show a statistically significant reduction in DCB usage following PTC withdrawal. There are several ethical implications to these findings, primarily highlighting patient beneficence and justice. Moving forward, it will be important to determine if this shift in treatment is owed to other treatment strategies such as BA, BA and atherectomy, BA and bare-metal stents (BMS), or BA and drug-eluting-stents (DES). The next steps should also include determining procedure costs and comparing readmission rates.

## Introduction

Balloon angioplasty (BA) and stenting - drug-eluting stents (DES) and bare-metal stents (BMS) - have long been the mainstays of endovascular therapy in peripheral arterial disease (PAD). In recent years, the rise of drug-coated balloons (DCBs) has revolutionized care, with multiple clinical trials showing superiority over BA in maintaining primary patency and freedom from target lesion revascularization (TLR) [1-12]. Some procedures with longer or multiple target lesions require multiple DCBs for treatment [1-5]. The increasing use of DCBs has gone hand in hand with an increasing body of positive clinical data and has also been supported by favorable reimbursement [1,2]. With the recent drop of the add-on payment for DCBs, known as pass-through code (PTC), a barrier for their use and consequently reduced therapy adoption in PAD may have arisen. We assess if this affected physicians' behavior and hospital administration towards stocking and using DCBs. Historically, products have decreased utilization because of lack of reimbursement - transcatheter aortic valve replacement (TAVR) is one such example [1,2]. We inquired if and how the change in DCB reimbursement impacted PAD patient care. To ensure optimal patient management, it is crucial to know how strategies and possible outcomes might shift and work towards a consensus between industry, physicians, and payers to provide an optimal foundation to facilitate the best care for patients. We hypothesized that the decrease in DCB reimbursement to hospitals (withdrawal of PTC) would disincentivize physicians to use DCBs, leading to altered treatment for PAD, including increased atherectomy usage and increased stent usage (BMS and DES).

## Materials and methods

Study design

This retrospective study observed and analyzed DCB utilization in 2017 and 2018 at a single interventional cardiology center in Raleigh, North Carolina. This study was approved by the Institutional Review Board at the University of North Carolina at Chapel Hill.

Inclusion and exclusion criteria

The patient cohort included individuals treated for peripheral arterial disease (PAD) involving the superficial femoral artery (SFA) or popliteal artery (POP) from July 1, 2017, to June 30, 2018. Patients who did not receive management for PAD by one of our institution's interventional cardiologists were excluded from the study.

Data collection

Subjects were identified using the International Classifications of Diseases 10 codes for PAD. Subjects were separated into two groups: July 1, 2017, to December 31, 2017 - with PTC - prior medical billing reimbursement (Group 1) and January 1, 2018, to June 30, 2018 - without PTC - markedly reduced medical billing reimbursement (Group 2). Data collected included demographics (age, sex), comorbidities (hypertension, hyperlipidemia, diabetes mellitus, coronary artery disease history, cerebrovascular accident history, tobacco use), number of DCBs used in patients, lesion location (SFA or POP), other lesional data (level of stenosis before BA, residual stenosis, length and diameter of reference vessel), DCB data (brand, diameter, length) if atherectomy was used, and its type (orbital, rotational, directional, laser) if stents were used and if they were drug-eluting, and if intravascular ultrasound (IVUS) was used and the characteristics of its plaque and imaged vessel's size.

Statistical analysis

Two-tailed t-tests assuming equal variance were used to analyze DCB usage and stent (DES and BMS) usage. DCB and stent data were expressed as means with standard deviations. The variable “atherectomy usage” was presented as percentages with standard deviations and analyzed by a Chi-square test with Yates correction. Demographics analyses were performed using two-tailed t-tests for continuous variables and Chi-square analysis with Yates correction for parametric variables. Statistical significance was defined as p<0.05.

## Results

Of the 486 subjects identified for the study, 389 met the inclusion criteria. The patient demographics and comorbidities did not differ before and after PTC withdrawal in 2017 and 2018, respectively (Table 1).

**Table 1:** Demographics and Comorbidities CAD: coronary artery disease; CVD: cardiovascular disease

	2017	2018	p-value	Chi-square
Number of Patients	209	180	N/A	N/A
Age (years)			0.0633	N/A
Mean	67.17	69.48		
Standard Deviation	12.69	10.18		
Gender			0.7106	0.1377
Male	113	93		
Female	96	87		
Hypertension			0.6505	0.2053
Yes	184	162		
No	25	18		
Unknown	0	0		
Hyperlipidemia			0.0586	3.578
Yes	158	151		
No	51	29		
Unknown	0	0		
Diabetes Mellitus			0.5889	0.2921
Yes	120	98		
No	88	82		
Unknown	1	0		
CAD/CVD			0.6727	0.1784
Yes	132	109		
No	77	71		
Unknown	0	0		
Tobacco Use			0.6769	1.523
Yes	64	45		
No	38	35		
Former	105	98		
Unknown	2	2		

**Figure 1:**
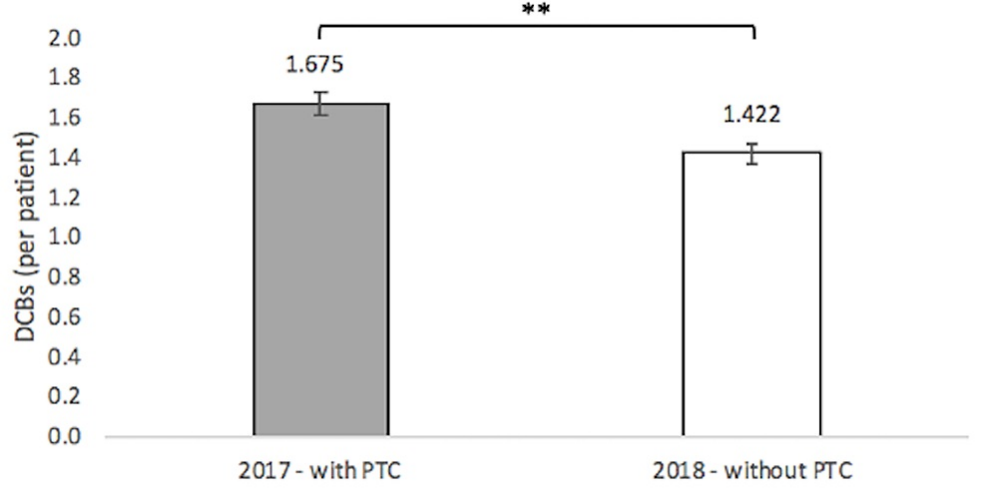
Drug-Coated Balloons Used With and Without Pass-Through Code

There was a 15.07% reduction in DCB usage without PTC (p<0.01) (Figure 1). 350 DCBs were used for the 209 patients sampled with the PTC in 2017 (1.675 DCBs per patient) with a standard deviation of 0.05466 DCBs. 256 DCBs were used in the 180 patients sampled without the PTC in 2018 (1.422 DCBs per patient) with a standard deviation of 0.05223 DCBs.

**Figure 2:**
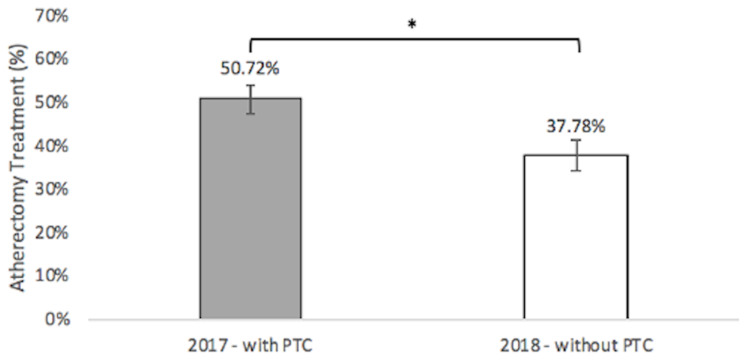
Atherectomy Treatment With and Without Pass-Through Code

There was a 25.51% reduction in atherectomy usage without PTC, with a 𝝌2 value of 6.550 (p<0.05) (Figure 2). One-hundred six of 209 patients were treated with atherectomy with the PTC in 2017 (50.72%) with a standard deviation of 3.467%. Sixty-eight of 180 patients were treated with atherectomy without the PTC in 2018 (37.78%) with a standard deviation of 3.624%.

**Figure 3:**
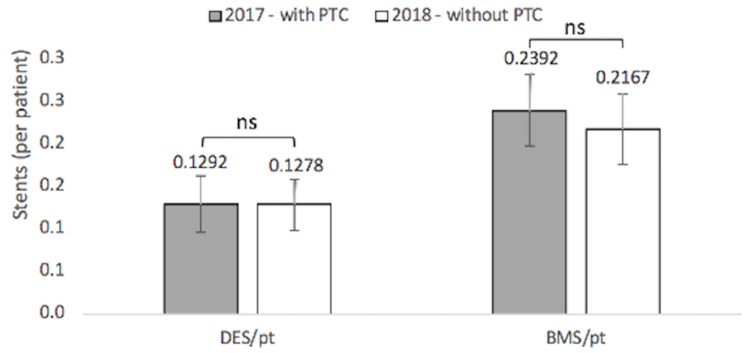
Drug-Eluting Stents and Bare-Metal Stents Used With and Without Pass-Through Code

Twenty-seven DESs were used in the 209 patients sampled with the PTC in 2017 (0.1292 DES per patient) with a standard deviation of 0.03306 DESs. Twenty-three DESs were used in the 180 patients sampled without the PTC in 2018 (0.1278 DES per patient) - a 1.091% reduction - with a standard deviation of 0.04237 DESs. 50 BMSs were used in the 209 patients sampled with the PTC in 2017 (0.2392 BMS per patient) with a standard deviation of 0.03054 BMSs. Thirty-nine BMSs were used in the 180 patients sampled without the PTC in 2018 (0.2167 BMS per patient) - a 9.433% reduction - with a standard deviation of 0.04039 BMSs. 63.16% of DCB-treated patients in 2017 and 65.56% of DCB-treated patients in 2018 were not treated with any stents. These data are speculative only as they are not to statistically significant levels (Figure 3).

## Discussion

Following the withdrawal of PTC on January 1, 2018, there was a statistically significant 15.07% reduction in DCB usage, which supports the first hypothesis that there would be a reduction in DCB usage in response to PTC withdrawal. The reduction in DCB usage showed clear physician responsiveness toward reducing DCB reimbursement and a shift toward BA treatment. This is relatively unsurprising; however, the long-term implications regarding patient care are unclear [13,14]. Given the superiority of DCBs over BA in improving TLR and maintaining primary patency, several ethical principles, including patient beneficence and justice, are called into question [15]. Future studies should consider following the trends for the need for revascularization and claudication rates in patients managed by interventional cardiologists before and after PTC withdrawal to evaluate for discrepancies [16,17].

There was also a statistically significant 25.51% reduction in atherectomy usage after PTC withdrawal, which supports the hypothesis that there would be altered atherectomy treatment; however, interestingly, it contradicts the second hypothesis that there would be an increase in atherectomy usage in response to the withdrawal of PTC. However, this contradiction supports a shift toward using BA to manage PAD and, therefore, should have been anticipated before the study's initiation.

There was a 9.433% reduction in BMS usage and a 1.091% reduction in DES usage following PTC withdrawal. However, these values did not reach statistical significance; we failed to reject the third null hypothesis. The static findings of DES and BMS usage suggest that stent usage may be unaffected by the change in DCB reimbursement. It should be noted that the sample size of subjects for these groups was relatively small. While the reduction in BMS usage did not reach statistical significance, it may be clinically relevant. The reduction in DES usage was low compared to BMS usage and is less likely to be clinically relevant.

In a world increasingly focused on the value of care, this study provides an analysis of the impact of DCB reimbursement and its utilization in treating PAD. Furthermore, it will give a framework to interpret changes in procedural cost and patient outcomes. One major limitation of this study includes its single-center nature, which limited our sample sizes. While we saw reductions in DES and BMS usage after PTC withdrawal, our observations were not to statistically significant levels. However, a multi-center analysis could provide more insight into these changes. Additionally, we could not analyze the effects of PTC withdrawal on BA utilization changes, which should be analyzed in future studies.

## Conclusions

The findings of this study show a statistically significant reduction in DCB usage following PTC withdrawal. There are several ethical implications to these findings, primarily highlighting patient beneficence and justice. Moving forward, it will be important to determine if this shift in treatment is owed to other treatment strategies such as BA, BA and atherectomy, BA and BMS, or BA and DES. The next steps should also include determining procedure costs and comparing readmission rates.

## References

[1] Tepe G, Laird J, Schneider P (2015). Drug-coated balloon versus standard percutaneous transluminal angioplasty for the treatment of superficial femoral and popliteal peripheral artery disease: 12-month results from the IN.PACT SFA randomized trial. Circulation.

[2] Krankenberg H, Tübler T, Ingwersen M (2015). Drug-coated balloon versus standard balloon for superficial femoral artery in-stent restenosis: the randomized femoral artery in-stent restenosis (FAIR) trial. Circulation.

[3] Cao S, He T, Xie J, Feng H, Liu K, Qu B, Wu X (2021). Drug-coated balloon angioplasty versus balloon angioplasty for treating patients with in-stent restenosis in the femoropopliteal artery: a meta-analysis. Medicine (Baltimore).

[4] Klumb C, Lehmann T, Aschenbach R, Eckardt N, Teichgräber U (2019). Benefit and risk from paclitaxel-coated balloon angioplasty for the treatment of femoropopliteal artery disease: a systematic review and meta-analysis of randomised controlled trials. EClinicalMedicine.

[5] Zhu Y, Liu K, Kong X (2021). Comparison of drug-coated balloon angioplasty vs. drug-eluting stent implantation for drug-eluting stent restenosis in the routine clinical practice: a meta-analysis of randomized controlled trials. Front Cardio Med.

[6] (2018). ClinicalTrials.gov: Lutonix DCB versus standard balloon angioplasty for treatment of below-the-knee (BTK) arteries. https://clinicaltrials.gov/ct2/show/NCT01870401.

[7] (2018). ClinicalTrials.gov: Randomized trial of IN.PACT Admiral® drug coated balloon vs standard PTA for the treatment of SFA and proximal popliteal arterial disease (INPACT SFA I). https://clinicaltrials.gov/ct2/show/NCT01175850.

[8] (2018). ClinicalTrials.gov: Stellarex DCB versus standard balloon angioplasty for treatment of below-the-knee (BTK) arteries (ILLUMENATE-BTK). https://clinicaltrials.gov/ct2/show/NCT03175744.

[9] (2022). ClinicalTrials.gov: Elutax-SV drug-eluting balloons for below-the-knee treatment (Apollo). https://clinicaltrials.gov/ct2/show/NCT02539940.

[10] (2022). ClinicalTrials.gov: First-in-man evaluation of a novel, microcrystalline paclitaxel coated balloon for treatment of femoropopliteal artery disease (PAX-r) (PAX). https://clinicaltrials.gov/ct2/show/NCT02145065.

[11] (2022). ClinicalTrials.gov: RANGER™ paclitaxel coated balloon vs standard balloon angioplasty (RANGER II SFA). https://clinicaltrials.gov/ct2/show/NCT03064126.

[12] (2022). ClinicalTrials.gov: Study comparing legflow versus bare balloon angioplasty for treatment of atherosclerotic disease. https://clinicaltrials.gov/ct2/show/NCT02710656.

[13] van Liebergen RA, Piek JJ, Koch KT, de Winter RJ, Lie KI (1998). Immediate and long-term effect of balloon angioplasty or stent implantation on the absolute and relative coronary blood flow velocity reserve. Circulation.

[14] Ueno K, Morita N, Kojima Y (2019). Safety and long-term efficacy of drug-coated balloon angioplasty following rotational atherectomy for severely calcified coronary lesions compared with new generation drug-eluting stents. J Interv Cardiol.

[15] Cameron AA, Laskey WK, Sheldon WC (2004). Ethical issues for invasive cardiologists: society for cardiovascular angiography and interventions. Cathe Cardio Interv.

[16] Beckman JA, Schneider PA, Conte MS (2021). Advances in revascularization for peripheral artery disease: revascularization in PAD. Circ Res.

[17] Deloge C, Boesmans E, Van Damme H, Defraigne JO (2018). Revascularization of the superficial femoral artery with paclitaxel-coated balloon for claudication. Acta Chir Belg.

